# Fundamental Movement Skills and Accelerometer-Measured Physical Activity Levels during Early Childhood: A Systematic Review

**DOI:** 10.3390/children7110224

**Published:** 2020-11-11

**Authors:** Alexandra Dobell, Andy Pringle, Mark A. Faghy, Clare M. P. Roscoe

**Affiliations:** Human Sciences Research Centre, College of Science and Engineering, University of Derby, Derby DE22 1GB 1, UK; a.pringle@derby.ac.uk (A.P.); m.faghy@derby.ac.uk (M.A.F.)

**Keywords:** fundamental movement skills, physical activity, balance, early childhood

## Abstract

Early childhood is a key period for children to begin developing and practicing fundamental movement skills (FMS), while aiming to perform sufficient physical activity (PA). This study reviews the current evidence for the levels of achievement in FMS and PA measured using accelerometers among 4–5-year-old children and examines differences by gender. This review was conducted using the PRISMA framework. Keyword searches were conducted in Pubmed, Medline, Google Scholar and SPORTDiscus. Inclusion criteria included age: 4–5 years old; FMS measurement: Test of Gross Motor Development 2 and 3; PA measurement: objective methods; balance measurement: static single limb; study design: cross-sectional observational/descriptive, randomised control trials, intervention studies; language: English. Twenty-eight articles from twenty-one countries met the inclusion criteria and were split into either FMS and PA articles (*n* = 10) or balance articles (*n* = 18). Three articles showed children achieving 60 min of moderate to vigorous PA per day, two articles demonstrated significant differences between girls’ and boys’ performance of locomotor skills and five reported locomotor skills to be more proficient than object control skills at this age for both genders. Balance was measured in time (*n* = 12), points score (*n* = 3) or biomechanical variables (*n* = 3), displaying heterogeneity of not only measurement but also outcomes within these data, with static single limb balance held between 6.67 to 87.6 s within the articles. Four articles reported girls to have better balance than boys. There is little conclusive evidence of the current levels for FMS, PA and balance achievement in young children 4–5 years of age. The academic literature consistently reports low levels of FMS competence and mixed evidence for PA levels. Inconsistencies lie in balance measurement methodology, with broad-ranging outcomes of both low and high achievement at 4–5 years old. Further research is required to focus on increasing practice opportunities for children to improve their FMS, increase PA levels and establish sufficient balance ability. Consistent and comparable outcomes during early childhood through more homogenous methodologies are warranted.

## 1. Introduction

Early childhood (4–5 years) is a critical time to develop health behaviours that are subsequently used throughout the lifespan and are important in reducing the likelihood of disease and illness during both childhood and beyond [1,2]. Among these behaviours are recommended levels of physical activity (PA). Ensuring children are sufficiently active is key to positive physical, cognitive and psychosocial health in children [1].

In the UK, government guidelines have been established and recommend that young children up to five years of age should aim to achieve 180 min of PA per day, with at least 60 min of this being moderate to vigorous PA (MVPA) [3]. This guideline is mirrored by multiple international governments, including Canada [4] (60 min of energetic play) and Australia [5]., Although the World Health Organization (WHO) does not provide guidelines for under 5 years of age, it recommends that children aged 5 years and above should achieve at least 60 min of MVPA each day, with additional PA at this level providing additional benefits [6]. Measurement of PA during early childhood is challenging, but essential to understanding how we can improve health in young children. Objective assessment such as accelerometry and pedometry offer the collection of continuous PA, without researcher or parental burden to report activity for young children [7,8]. These measures have been identified as more reliable and accurate compared to subjective measures over extended periods of assessment [9,10] and are now recognised as a preferred method. Accelerometer type, placement on the body and the cut points and epochs employed by researchers have seen great variation in the literature, and can affect overall PA outcomes, questioning the validity and objectivity of accelerometry [11]. Identifying these variations or if there are more commonly made choices by researchers to create an overall picture of how PA is objectively examined in young children is important. Further to this, there is currently not a consensus if young children achieve the amount of PA guidelines recommend when measured using accelerometry.

Over the last 20 years there have been multiple studies examining PA behaviours and more recently more studies examining fundamental movement skills (FMS) and their relationship with PA. Fundamental movement skills are the building blocks to more complex movement patterns and are made up of object control skills such as throwing, catching, kicking and stationary bounce, locomotor skills such as running, jumping, hopping and skipping and stability skills such as static and dynamic balance [12,13]. Each element is equally important to increasing overall motor competence (MC) and gross and fine motor skills, in addition to performing day to day activities and postural control [14,15,16,17]. Creating an environment for developing fundamental movement skills (FMS) and motor competence (MC), including adequate skill practice opportunities, is vitally important to sustained involvement in and achievement of PA for children of this age and onwards [18]. At young ages (3–5 years old), children should be encouraged to perform MVPA to help develop these FMS and higher levels of motor competence [19]. Understanding the current prevalence of FMS competency during early childhood is important to identifying areas of development for both research and intervention.

The Test of Gross Motor Skills 2 and 3 (TMGD-2 and -3) [20,21] are frequently adopted methods for the assessment of MC for young children across the globe. In 2018, Logan at al. [13] reported that it was the most widely used tool (51%) in a review of 124 studies examining FMS measurement and terminology. This is further supported by Klingberg et al. [22] noting the popularity of the TGMD for measuring FMS in preschool children. The strengths of the TGMD protocols and assessment include the reliability of the process-based scoring system, where two attempts of each skill are scored with between three and five performance criteria for the 12 or 13 skills in the TGMD-2 and TGMD-3, respectively, while also providing good reliability and validity as a measurement tool [23]. These batteries also employ measurement of skills commonly seen in play and sport performance [24], which are vital for PA participation throughout childhood and the lifespan [25].

Balance and stability are critical parts of FMS [26], yet are rarely examined in large-scale studies alongside locomotor and object control FMS [27]. Interestingly, the MC assessment tools, TGMD-2 and TGMD-3, are both without elements of static or dynamic balance assessment [20,21]. Although other assessment batteries have been developed, such as the Movement Assessment Battery for Children [28] and Bruininks–Oseretsky Test of Motor Proficiency [29], that both examine balance in addition to fine motor skills, the TGMD batteries remain as one of the most popular forms of assessment, especially for gross motor development [13].

Given its importance, various studies have examined the balance ability of young children in separate studies, using tools such as the paediatric balance scale (PBS) [30]. Hardy et al. (2010) [31] previously stated that stability and balance ability has ceiling effects during early childhood and, by the age of four years, children have sufficient mastery of dual stance balance [32,33], therefore, the inspection of single limb balance can be employed to explore the development of more complex balance ability at these early years. Some studies have stated that during the ages of four to five years, there is a large natural development of balance ability [34,35], and therefore discount the need for measurement. This matches findings by both Chow and Chan [36] and Krombholz [37], who reported continually increasing balance ability as children aged. Nonetheless, static balance is known to be an important precursor to locomotor activities [38] and inspection of balance ability in young children will help inform both future research and interventions, allowing young children to achieve better overall FMS and explore the association between locomotor, object control and balance skills. Physical activity and FMS are clearly interrelated and stand to give researchers and practitioners an important overview of key behaviours and skill development in young children. Currently, locomotor, object control and balance competency and PA behaviours have not been summarised for children of 4–5 years, a key age for both balance development [31] and the start of formal education in the UK [39], where children are asked and expected to use such skills on a regular basis. Although there are a wide range of assessment methodologies employed to test and measure levels of MC, FMS, PA and balance in children [23], this review will focus on the TGMD-2 and -3 protocols for locomotor and object control skills, due their consistent popularity and dedicated focus of measuring gross motor development, designed for typically developing children 3–10.9 years of age [20,21]. Balance ability is observed through dedicated balance studies of this age group. Therefore, the aim of the current systematic review is to summarise the methods used and outcomes achieved during assessment of the current levels of achievement of locomotor and object control FMS, PA and balance competency in 4–5-year old children.

Three main objectives have been identified for the purpose of this review with young children 4–5 years of age: (1) observe the locomotor and object control skill competency of young children measured via the TGMD batteries, (2) observe current physical activity achievement in young children and how these are measured using objective methods, (3) identify common static balance measurement methods and achievement in young children.

## 2. Materials and Methods

### 2.1. Protocol and Registration

This review was registered with PROPSERO (registration number CRD42020181666) in June 2020. The review protocol can be found by searching this number on the PROSPERO website or using the address: https://www.crd.york.ac.uk/prospero/display_record.php?RecordID=181666.

### 2.2. Study Selection Criteria

A systematic review of the literature was conducted using the Preferred Reporting Items for Systematic Reviews and Meta-Analyses (PRISMA) framework [40] (please see Appendix A, Table A1 for checklist) to identify all English language, peer-reviewed articles published between January 2000 and April 2020. Although articles must have been published in the English language, they could originate from across the globe, as this allowed for a broader picture of achievement in children aged 4–5 years. For both searches, observation studies, prospective cohort studies, baseline studies, intervention studies (if pre-intervention data were available) and validity studies were included, however, review articles were excluded from analysis. 

The literature met the following criteria for the series of articles examining FMS and PA: participants of age 4–5 years; examined FMS measured by the TGMD-2 or -3; examined PA measured via objective measures; reported levels of MVPA over an hourly or daily period; data were collected at baseline or as part of an observational study, data were collected from typically developing and typical weight children without disability or developmental delay. The literature met the following criteria for the series of articles examining balance: participants of age 4–5 years; examined static balance in single-leg stance; data were collected at baseline or as part of an observational study, data were collected from typically developing and typical weight children without disability or developmental delay.

If articles included data for other age groups, then data must have been explicitly reported for 4–5 year olds for the article to be eligible for inclusion. Articles must have explicitly reported data for typically developing and typical weight children if a comparison between another group with a disability, developmental delay or obesity was being examined. This was applicable for both the FMS and PA searches and balance searches.

### 2.3. Search Strategy

**Figure 1:**
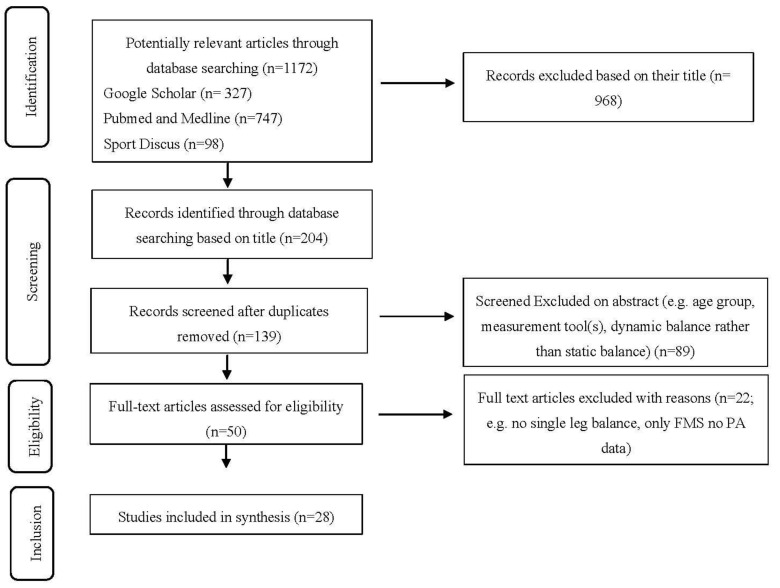
Combined searches Preferred Reporting Items for Systematic Reviews and Meta-Analyses (PRISMA) flowchart.PA: physical activity; FMS: fundamental movement skills.

**Figure 2:**
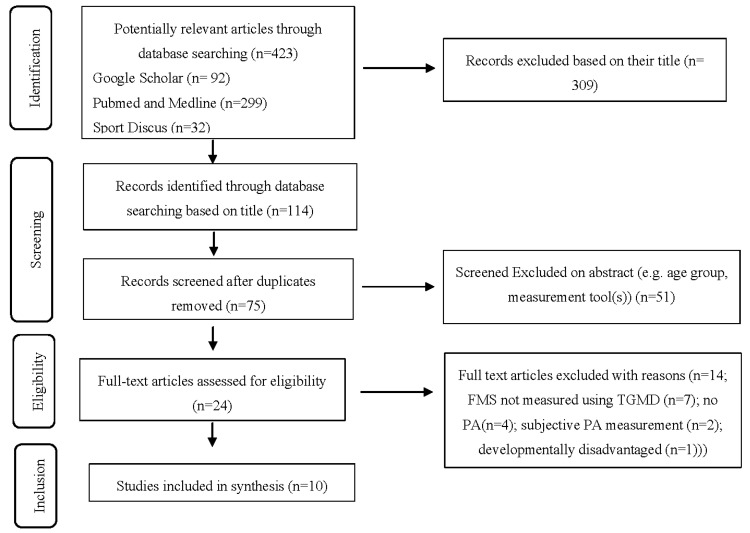
Locomotor, object control and physical activity (PA) searches PRISMA flow chart. TGMD = Test of Gross Motor Development.

**Figure 3:**
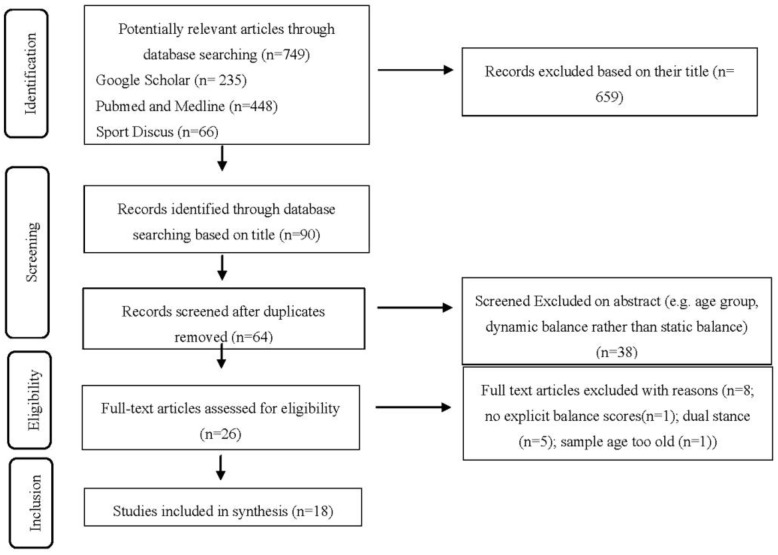
Balance searches PRISMA flow chart.

PubMed/Medline, Google Scholar and SPORTDiscus were searched up to 30 April 2020 using the following key words within the titles: fundamental movement skills, physical activity and children for the FMS/PA articles. For example, Google scholar was searched using the terms “allintitle: ‘physical activity’ ‘children’ ‘fundamental movement skills’”. The balance literature was searched for using a combination of the keywords: balance, childhood, early childhood, children and young children. For example, Pubmed and Medline were searched using the term “(balance [Title]) AND childhood [Title]”. A screening of titles according to the criteria was completed. Subsequently, any duplicates from separate search engines were removed. A further screening of the abstract was undertaken according to the inclusion criteria and, if it was not clear if an article met the inclusion criteria at this stage, it was included in the full text screen. Full text articles were then assessed for eligibility, a visual representation of the search strategy can be observed in Figure 1, Figure 2 and Figure 3. Ten percent of the original search sample was randomly allocated and examined by a second researcher (CR), confirming or disagreeing with the first researcher’s (AD) decision. Initially, disagreement was recorded among 7% (*n* = 1) of the articles. Discussion was held between the researchers to reach a consensus on inclusion of specific articles, which matched the first author’s decision.

### 2.4. Data Extraction and Synthesis

For the articles that met the criteria, the following data were extracted for all paper types: author(s), year of publication, country of origin, setting, sample size for age 4–5 years, mean age of participants, study design (observational, randomised controlled trial, non-randomised trial), outcome measure(s) and overall findings related to FMS and PA achievement or balance ability. 

#### 2.4.1. Locomotor, Object Control and PA Articles

For FMS and PA articles, outcome measure(s) included: FMS assessment battery, accelerometer type and location. FMS competency was reported as per individual article, for example, as a gross motor quotient or as a raw score. Studies included in the current review used the full form of 12 or 13 items to assess motor competence and reported the raw scores out of 96 or 100 points, respectively. However, some studies use shortened protocols, and these studies were still included, resulting in not all studies having full scoring.

MVPA has been recognised as being particularly beneficial for health during childhood and throughout the lifespan [41,42] and therefore this was considered the key measurement during this review. Daily and hourly MVPA levels were reported and gender differences were reported where available. Information on the type of accelerometer, placement on the body, the cut points and epochs employed was also deemed important.

#### 2.4.2. Balance Articles

For balance articles, outcome measure(s) included: balance assessment conditions and measurement tool(s). Where reported, biomechanical aspects of balance, including centre of pressure (COP) area and length and time spent (measured in seconds) in a single-leg stance, were recorded as balance outcomes. Further to this, single-leg balance tests from test batteries, including the Bruininks–Oseretsky Test of Motor Proficiency Second Edition [29] and Movement Assessment Battery for Children [28] that use a numerical score of balance, were reported for this element of the review.

### 2.5. Study Quality Assessment

The mixed methods appraisal tool (MMAT) [43] was used to assess the quality and risk of bias within the studies included in the review. The studies were screened using two initial questions before being appraised according to five criteria according to their study design category. The categories included in this study included quantitative randomised controlled trials, quantitative non-randomised studies and quantitative descriptive studies. Questions/criteria were answered using; yes, no or cannot tell. For any “yes” answers a paper received one mark, any “no” or “cannot tell” would receive no marks, thus the maximum score for an article was 7. Quality score was used to indicate the strength of the evidence from the individual studies but was not used to determine their inclusion or exclusion within the review.

### 2.6. Analysis

Analysis for both the FMS and PA articles and balance articles was of a narrative approach due to the lack of heterogeneity of the data collected and multiple methods used.

## 3. Results

### 3.1. Study Selection

Overall, 1172 articles (including possible duplicates) were identified using the key word search across three search engines. Following this, 986 articles were excluded based on their title. Following removal of duplicate articles, a further 89 articles were excluded following examination of the abstract. Fifty articles were assessed for full text eligibility and 28 articles were included in the final analysis. The most common reasons for exclusion included age group, i.e., being too old, subjective PA measures, alternative FMS assessment batteries to TGMD, dynamic balance measurement and static dual stance balance. These are reported in Figure 1, Figure 2 and Figure 3., which shows how the study selection was completed for the study, FMS and PA articles and balance articles, respectively.

### 3.2. Origin and Participants

Of the 28 articles analysed, there were multiple countries of origin for both the FMS and PA articles and balance articles. For FMS and PA, of the 10 articles used in the analysis, there was a total of six different countries the articles originated from. This included: three articles from Australia, two from the UK and USA and one from Ireland, Norway and Canada. For the balance literature, the 18 articles originated from 15 different countries including: two articles from Serbia, Korea and the USA and one from Spain, Indonesia, Belgium, Australia, Iran, Japan, China, Brazil, Romania, Greece, Ireland and Singapore.

The total number of participants for the FMS and PA articles combined was 1514. The average number of participants was 151 and ranged from 46 to 376. The total number of participants for the balance-related articles combined was 5036, the average number of participants was 280 and this ranged from 15 to 3575 participants. Therefore, the overall participation number for this review is 6550 participants with an average age of 4.7 years.

Of the FMS and PA articles, 90% reported the gender of the participants. The sample consisted of 1485 participants, 54% were boys and 46% girls. Of the balance articles, 78% reported the gender of the participants, (*n* = 4876), with 51% of them boys and 49% girls.

### 3.3. Study Quality Assessment

Most of the articles included were of high quality according to the MMAT. Using the MMAT, 58.6% (17 articles) of the studies were highly rated, meeting all seven criteria set out by the tool. A further 24.1% (seven articles) met six criteria, 13.8% (four articles) met five criteria and just 3.5% (one article) met four of the criteria. Four articles were assessed under the quantitative randomised controlled trial criteria, one article under the quantitative non-randomised criteria and the remaining 24 under quantitative descriptive criteria. Individual study scores can be found in Appendix B (Table A2).

### 3.4. Locomotor and Object Control Proficiency

Of the 10 articles collated for analysis (Table 1), all had employed the use of the full or partial TGMD-2 or TGMD-3 protocols as stipulated by the criteria. As these protocols had a different total score, due to allowing the inclusion of research that had not employed the full protocols or used a different scoring system, such as the Children’s Activity and Movement in Preschool Study Motor Skills Protocol (CMSP), a meta-analysis could not be conducted.

Of the ten articles reviewed, three articles used the full TGMD-2 protocol and scored this against the TGMD-2 criteria [44,45,46]. A further three articles used a version of the TGMD-2 protocol adapted for use within individual studies [47,48,49]. This included using specific skills, removal of unwanted or unwarranted skills and addition of more relevant skills related to the study outcomes or cultural differences. Foweather et al. [50] used the full TGMD-2 protocol and scored this using the CMSP. Three articles employed the use of the TGMD-3 protocol, which is an updated version of the TGMD-2 protocol [19]. Two of these articles used the full protocol, which scores 13 skills (six locomotor and seven object control skills) [51,52], while Nilsen et al. [53] used a partial protocol.

A common theme found between the motor proficiency performance of the children was the majority of authors reporting locomotor skills being performed with more competency than object control skills at this age [46,47,50,53] and, similarly, both Duff et al. [47] and Jones et al. [48] found running to be the most proficient individual skill at this age over any other individual locomotor or object control skill tested. On the other hand, only Palmer et al. [51] found the opposite in their baseline assessments, with object control skills being performed with more proficiency.

Where gender differences were reported, it was found that girls performed locomotor skills with more proficiency than boys [45,49], however, both authors reporting this found that there was no significant difference between the object control or total raw scores between genders. On the contrary, Webster et al. [52] found boys performed object control skills with higher proficiency than girls. When relationships with PA were assessed, Nilsen et al. [53] found a significant and positive relationship between MVPA levels and locomotor and object control skills, while Roscoe et al. [49] reported FMS mastery did not influence the PA level achieved.

### 3.5. PA Levels

Of the FMS and PA articles reviewed (*n* = 10) (Table 1), accelerometry was used to assess PA, with no other objective method used to measure PA, such as pedometry. Seven articles assessed PA using hip-based accelerometry and ActiGraph models including the ActiGraph 7164, GT1M and GT3X (ActiGraph LLC, Pensacola, FL, USA), [44,45,47,48,50,51,52,53]. Wasenius et al. [46] used an Actical accelerometer, the position of the accelerometer was not stated. Roscoe et al. [49] was the only article to use wrist-based accelerometry and a Geneactiv accelerometer. Roscoe et al. [49] also used the Roscoe et al. [54] Geneactiv, wrist-based cut points to classify activity of the children. Further to this, three articles used Evenson et al. [55] cut points [44,51], one used Pate et al. [56] cut points [52], one used Adolph et al. [57] cut points [46], one used Sirad et al. [58] cut points [48], one used Janssen et al. [59] cut points [50], one used Pate et al. [60] cut points [47] and one article used both Reilly et al. [61] and Sirad et al. [58] cut points to classify activity. For the measurement of epoch length, one article used 1 s epochs [53], one article used 5 s epochs [50], two articles used 10 s epochs [47,49], five articles used 15 s epochs [44,46,48,51,52] and finally one article used 1 min epochs [45]. Therefore, a wide breadth of different cut points and epoch lengths was used in identifying PA behaviours of the participants in the studies.

Wear time varied between two days [48] and 14 days [53], with the most commonly reported length being seven days [45,46,50,51,52]. Barnett et al. [44] reported eight days’ wear, while Roscoe et al. [49] reported four and Duff et al. [47] five days of wear. However, out of the eight articles that stipulated an acceptable wear time for analysis, six articles required three days of wear [45,46,47,49,50,52] and the remaining two articles required four days of wear time [44,53].

Webster et al. [52] reported the highest levels of MVPA with 102 min per day, while Cliff et al. [45] only reported 23 min of MVPA per day. Of the articles reporting hourly MVPA, this varied between 13.6 min to 22.42 min per hour. Jones et al. [48] reported that 7% of the children’s day at preschool was spent in MVPA level.

### 3.6. Balance Proficiency

Balance articles (Table 2) were limited due to the inclusion of static balance, however, there was still sufficient evidence for analysis of this literature. Of the balance articles, 18 were used for data extraction and synthesis. There was not a common assessment method, therefore, a meta-analysis could not be undertaken.

All articles included static balance tests that required the children to balance on one leg. Some articles (*n* = 11) reported this simply on a firm surface with eyes open [32,34,62,63,64,65,66,67,68,69,70]. Stankovic and Radenkovic [71] also asked children to stand on a single leg on a firm surface, repeating this with their eyes closed. Venetsanou and Kambas [35] and Eshaghi et al. [72] used balance beams in their methods. Meanwhile, other articles reported static balance on unstable surfaces such as foam [73,74,75] with or without visual aid (eyes closed). The articles that employed the use of foam surfaces, balance beams and closing of the eyes all reported a reduction in performance in a single-leg stance during these conditions. Barefoot and shod conditions were employed by Tan [76] and found no significant difference in the two conditions.

Twelve articles used time, in seconds, as the outcome measure for the single-leg tests [63,64,65,66,67,68,69,71,72,73,76]. The range of time the single-leg stance was maintained for on a hard surface with eyes open ranged from 6.67 s [63] to 87.6 s [65], with eyes closed, 6.9 s was the lowest outcome [71] with 33.62 s being the highest [73]. Three articles used point scores to measure the outcome of the balance, including Adamovic et al. [62], Guffey et al. [32] and Venetsanou and Kambas [35]. Meanwhile, the remaining three articles used biomechanical characteristics to measure balance [34,70,74], which are reported in Table 2.

Five articles reported gender differences between the children, as four articles reported girls having better balance than boys [34,35,69,71] and, conversely, Marin [67] reported boys to have better balance ability than girls. Four articles reported the effect of age on the children’s balance ability, with clear increases in time held [69], points scored [35] or reduction in sway velocity or centre of pressure movement recorded [34,74] as the children aged.

**Table 1:** Locomotor, object control and PA article descriptive results.

Author	Country	Setting	Sample Size	Mean Age (years)	Study Design	Outcome Measure(s)	Overall Findings—Relating to Baseline FMS and PA
Barnett et al. (2016) [44]	Australia	Home setting	127 (59 boys, 68 girls)	5 ± 0.1	Observational cohort study	TGMD-2 (full), Accelerometry MVPA levels @ hip, ActiGraph GT1M	(1) Most children were average or below their age recommended standard score for TGMD-2. (2) Children performed 52.8 min/day of MVPA.
Cliff et al. (2009) [45]	Australia	Preschool	46 (25 boys, 21 girls)	4.3 ± 0.7	Cross-sectional	TGMD-2 (full), Accelerometry MVPA levels @ hip, ActiGraph 7164 uniaxial	(1) Girls had a higher LOCO level and gross motor quotient than boys, but no gender difference OC with raw scores. (2) The sample overall spent 23 min/day in MVPA levels.
Duff et al. (2019) [47]	Ireland	Preschool	141 (71 boys, 70 girls)	3.9 ± 0.5	Cross-sectional baseline	TGMD-2 and Victorian FMS manual- run, vertical jump, throw and catch Accelerometry ActiGraph GT3x and GT1m @ Hip (only preschool time)	(1) Children were proficient in run (88.4%), but low across other skills assessed (4.9–18.5%). (2) 7.7 min/h MVPA on average over 3 h school day.
Foweather et al. (2015) [50]	England	Preschool	99 (52 boys, 47 girls)	4.6 ± 0.5	Cross-sectional observational	TGMD-2 scored with CMPS (total 138), Accelerometer ActiGraph GT3X@ waist	(1) Children completed more VPA at weekends vs. on weekdays. (2) On average children completed 89.4min MVPA/day. (3) Children had higher proficiency of LOCO than OC skills.
Jones et al. (2011) [48]	Australia	Preschool	97 (no gender data)	4.13	Cluster randomised controlled trial	TGMD-2 (5 skills; Run, Jump, Hop Catch, Kick), MTI 7164 ActiGraph accelerometer @ right hip	(1) 7% of time was spend in MVPA. (2) Children were most proficient in the run and least proficient in the hop.
Nilsen et al. (2020) [53]	Norway	Preschool	376 (196 boys, 180 girls)	4.7 ± 0.9	Cross-sectional observational	TGMD-3 (partial), Accelerometer ActiGraph GT3X @ right hip	(1) Children had higher competence in LOCO than OC skills. Total FMS 25.5/44. (2) 70min/day of MVPA. (3) Significant and positive relationship between MVPA levels and LOCO and OC skills.
Palmer et al. (2018) [51]	USA	Preschool	102 (63 boys, 39 girls)	4.4 ± 0.43	Randomised control trial	TGMD-3 (Full), Accelerometer ActiGraph GT3X@ waist	(1) 22.42 min/h as MVPA, with boys presenting more MVPA than LPA compared to girls. (2) Children were more proficient in OC than LOCO skills at baseline. (3) 19/100 was achieved for total FMS at baseline.
Roscoe et al. (2019) [49]	England	Preschool	185 (99 boys, 86 girls)	3.4 ± 0.5	Cross-sectional observational	TGMD-2 (no underhand roll, added skip), Accelerometer- Geneactiv @wrist	(1) None of the children achieved the PA recommendations and were inactive, average MVPA was 25 min/day. (2) Girls scored better in the LOCO skills, and boys scored better in the OC skills, no sig. diff. in total FMS. (3) FMS mastery level did not influence PA levels of the children. Children scored from 6–82 points for total FMS, an average 52/90.
Wasenius et al. (2018) [46]	Canada	Preschool	215 (117 boys, 98 girls)	3.65 ± 0.5	Cluster randomised controlled trial	TGMD-2 (full), Accelerometer Actical, omnidirectional	(1) At baseline LOCO skills were more proficient than OC between groups. (2) Children’s average GMQ was 37.7/96. (3) 25 min/h average PA performed by all children at baseline over 5 h wear.
Webster et al. (2019) [52]	USA	Childcare centres	126 (58 boys, 68 girls)	3.4 ± 0.5	Observational cohort study	TGMD-3(full), Accelerometer ActiGraph GT3X @ right hip	(1) 1.7 ± 0.6 h of MVPA per day. (2) Boys had better total TGMD-3 and OC scores than girls. (3) The average percentile for children 45.2 for overall FMS, or 37.7 points out of 100.

Key: PA = physical activity, FMS = fundamental movement skill, MVPA = moderate to vigorous physical activity, TGMD = Test of Gross Motor Development, LOCO = locomotor, OC = object control, CMPS= Children’s Activity and Movement in Preschool Study Motor Skills Protocol, LPA = Light Physical Activity, GMQ = Gross Motor Quotient.

**Table 2:** Balance article descriptive results.

Author	Country	Setting	Sample Size	Mean Age (years)	Study Design	Outcome Measure(s) for Single-Leg Balance	Overall Findings—Relating to Static Balance
Adamovic et al. (2015) [62]	Serbia	Testing at university	54 (29 boys, 25 girls)	5.24 ± 0.14	Longitudinal observational	Standing on one foot for 20 s: score from 0–2.	(1) Standing on one foot was the least developed balance skill of the children. (2) Average score was 1.5/2 with 34% (20 children) of the sample achieving the maximum of 2 points (10–12+ s)
Amelia et al. (2019) [63]	Indonesia	N/A	30 (no gender data)	5.5	Non-equivalent control group	Standing on single leg (trial on each leg) for up to 30 s, measured in seconds.	(1) Children were able to hold single-leg balance for 6.67 s on average at baseline.
An et al. (2009) [73]	Korea	Elementary school	18 (no gender data available)	5	Cross-sectional observational	Single-limb standing test; 4 conditions; firm surface: eyes opened and closed, foam surface: eyes open and closed. Measured in seconds.	(1) Children could hold a single-leg balance for 37.55 ± 21.11 s with a firm surface and eyes open. (2) When eyes were closed this decreased to 33.62 ± 21.60 s.
Cambier et al. (2001) [74]	Belgium	Primary School	73 (no gender data available)	4.5	Cross-sectional observational	Unilateral stance test, 4 conditions; firm surface: eyes opened and closed, consecutively on the left then right foot, measured using centre of gravity sway velocity.	(1) There was a higher sway velocity when eyes were closed on both feet, suggesting more movement in this position. (2) Between 4 and 5 years of age, the velocity of movement reduced.
Condon and Cremin (2014) [75]	Ireland	Mainstream primary schools	26 (11 boys, 15 girls)	4.5	Cross-sectional	Single-limb standing test; 3 conditions; firm surface: eyes opened and closed, foam surface: eyes open, measured in seconds.	(1) Standing with eyes open allowed children to achieve a higher time. (2) Eyes closed and foam surfaces disrupted children’s balance, foam being the greatest disrupter for young children.
De Oliveira et al. (2019) [64]	Australia	Primary School	511 (257 boys, 254 girls)	5.4	Cluster randomised control trial	One-leg balance: MABC-2, measured in seconds.	(1) At baseline children could hold a one-leg balance for an average of 16.75 s. (2) The intervention was found to have a positive effect.
Eshaghi et al. (2015) [72]	Iran	N/A	20 (9 boys, 11 girls)	6	Cross-sectional observational	One-leg balance eyes open and closed on ground and repeated on balance beam from BOTMP-2, measured in seconds.	(1) Term children could remain balanced on one leg on the ground for an average of 9.70 s. This decreased by 1 s with eyes closed. (2) On a balance beam, balance was further reduced.
Fujinaga (2008) [65]	Japan	Kindergarten	105 (51 boys, 54 girls)	5	Cross-sectional observational	One-leg standing test, measured by time, up to 120 s (seconds).	(1) Children on average achieved 87.6 ± 37.06 s in the single-leg stance.
Guffey et al. (2016) [32]	USA	Hospital paediatric clinic and day care	28 (no gender data)	3.54 ± 0.84	Cross-sectional observational	Paediatric Balance Scale component 9 (standing on one foot), scored 0–4.	(1) Standing on one foot was recognised as a harder task for children to perform from the PBS, however, it was generally mastered by the age of 4 years for 10 s.
Jiang et al. (2018) [34]	China	Public kindergarten	60 (30 boys, 30 girls)	4.5	Cross-sectional observational	Tekscan foot pressure measurement system, one foot eyes open, held for 10 s. Measured by envelope area (area), path length (length), maximum displacement in anteroposterior (forward–back) and mediolateral direction (left–right) of the centre of pressure.	(1) Girls had lower postural sway than boys. (2) There were no significant differences between balance measurements on one foot at ages 4 and 5 years. However, 5 year olds’ movements were consistently lower than 4 year olds, showing the increase in balance at this age.
Jung et al. (2017) [66]	Korea	N/A	11 (4 boys, 5 girls)	5.8 ± 1.2	Cross-sectional observational	One-leg standing test (OLST) non-dominant leg, measured in seconds.	(1) On average children stood in the single-leg position for 38.1 ± 20.8 s.
Marin (2012) [67]	Romania	Kindergarten	20 (9 boys, 11 girls)	4.5	Observational cohort study	The flamingo test for up to one minute, measured in seconds.	(1) 33.16 s was the average length of time children held the flamingo test for. (2) Boys (42.22 s) were much more proficient in holding the flamingo test than girls (24.09 s).
Moran et al. (2005) [68]	Brazil	Public school	136 (62 boys, 74 girls)	5	Cross-sectional	Single-leg stance test for 10 s.	(1) 60 (44%) of the children in the control group failed to hold the single-leg balance for 10+ s.
Latorre-Roman et al. (2017) [69]	Spain	Preschool	3575 (1816 boys, 1759 girls)	4.7 ± 0.93	Cross-sectional observational	Stork balance stand test, up to one minute, measured in seconds.	(1) At 4 years of age girls were more proficient than boys at balancing on their right leg. (2) On average children held the stork stance for 8.13 ± 7.81 s. (3) Between ages 4 and 5 years children’s balance improved from 7.40 ± 6.99 to 10.51 ± 8.84 s, showing the improvements at this age.
Stankovic and Radenkovic (2012) [71]	Serbia	Preschool	39 (26 boys, 13 girls)	5.5	Observational cohort study	Standing on one leg eyes open and standing on one leg eyes closed, measured in seconds.	(1) Children held their balance with eyes open for an average of 25.85 s, girls holding for longer than boys (28.8 vs. 22.9 s). (2) With eyes closed, 6.9 s was the mean score for all participants. Again, girls were able to hold this position for longer (7.3 vs. 6.5 s).
Tan et al. (2019) [76]	Sinagpore	N/A	23 (9 boys, 14 girls)	6.32 ± 0.27	Cross-sectional observational	One-leg balanc:- MABC-2, measured in seconds. Barefoot and shod conditions, up to 30 s tested.	(1) Children were able to hold the one-legged balance for 25.74 ± 5.778 s while barefoot and 25.04 ± 6.698 s when shod. These were not significantly different.
Venetsanou and Kambas (2011) [35]	Greece	Public preschools	283 (145 boys, 138 girls)	5.15 ± 0.45	Cross-sectional observational	BOTMP; standing on the preferred leg on the floor, standing on the preferred leg on a balance beam, standing on the preferred leg on a balance beam—eyes closed. Numerical point score.	(1) Girls scored consistently higher on all single-leg balance elements than boys. (2) The 54–59 month age group was significantly worse at balancing than the 60–65 month and 66–71 month age groups, showing clear progression at these ages.
Zumbrunn et al. (2012) [70]	USA	Test in laboratory	15 (9 boys, 6 girls)	6.17 ± 1.1	Evaluation study	Stand on one foot for 5 s, COPsd A/P, COPsd M/L, COPsd Res, COPmax A/P, COPmax M/L, COParea, ARD, path velocity, COPvel A/P, COPvel M/L, ARF	(1) COP elements correlated well with the BOT balance subtests.

BOTMP-2 = Bruininks–Oseretsky Test of Motor Proficiency-2, PBS = Paediatric Balance Scale, MABC-2 = Movement Assessment Battery for Children 2, COP = centre of pressure, ARD = Average radial displacement, ARF = average radial frequency.

## 4. Discussion

The aim of the current study was to review the current evidence and literature surrounding 4–5-year-old children’s FMS proficiency using the TGMD, PA levels and single-limb balance ability, whilst identifying the important variation in methodologies employed by researchers to reach these research outcomes. Low levels of locomotor and object control proficiency were found across the literature, in addition to a varied but still worrying picture around PA recommendations, further complicated by factors such as accelerometer wear time and cut points. Balance measurement was found to be common in early childhood but not combined with locomotor and object control assessments. Methodological differences were a key issue, leading to no conclusive evidence for balance ability. A total of 28 articles were identified through keyword database searches. Ten of these related to locomotor and object control FMS and PA, and a further 18 related to balance ability and measurement. Literature in the English language was available from around the globe and provided data on locomotor and object control, PA and balance during early childhood. Gender, skill type (locomotor vs. object control) and physical development from 4–5 years old appear to affect these variables.

### 4.1. Achievement

Where explicitly reported, total TGMD scores and specific locomotor and object control FMS skills were of low competence or below those expected for the children’s ages [44,46,47,51,52]. Authors reported standardised TGMD-2 scores [20], raw total scores and percentile scores across the literature. It should be noted that standardised, percentile and age equivalent scores are based on data from American samples only, and these data do not currently exist for other countries. Additionally, Roscoe et al. [49] reported that raw scores varied from 6 points to 82 points out of a possible 90 for total FMS, illustrating that even at young ages, a wide range of achievement is present. Although children aged 4–5 years are not expected to achieve mastery across FMS proficiency, it has been noted in the literature that this is a possibility by the age of six years and children should have begun to establish movements from four years of age [12,26]. Therefore, a greater emphasis should be placed on FMS tuition at these younger ages, such as interventions and programmes in care and educational settings focussing on FMS performance and development in early childhood to increase practice opportunities and the quality of teaching for young children. Practices such as these will ensure a larger number of children reach full potential in their motor development. It has already been established and commonly reported that higher MC ability will ultimately lead to better levels of PA engagement, overall habitual activity levels and thus better health behaviours, especially during middle childhood and onwards [18,77]. This is further supported by the Jones et al. [78] review that found a positive association between FMS, MVPA and total PA across 19 studies in the early years. Therefore, the argument to ensure purposeful development and practice of these skills at a young age is strong. This should be achieved through clearer guidelines, recommendations and training based on research outcomes for early years educators and care givers. Previous research has cited underprepared and under trained early years educators and care givers [79] to be a key issue leading to this low achievement in young children. As such, further work needs to be undertaken to enhance their preparedness to promote FMS, including an understanding of the barriers and facilitators impacting their work/engagement.

Fifty percent of the articles reported that children’s locomotor ability was better at ages 4–5 years than their object control skill ability [46,47,48,50,53], and thus could contribute to the performance of more MVPA. Locomotor skills generally require children to perform large gross motor movements which involve the whole body [26]. Object control skills tends to include elements of fine motor skills, such as gripping, and more complex processes, including hand–eye coordination, possibly explaining their lower achievement at this age. There was some repeated evidence for girls performing locomotor skills with higher proficiency than boys [44,49], this is commonly attributed to cultural and social gender norms, even at these young ages, where girls are more likely to take part in activities requiring more repetition of locomotor skills [80]. This conclusion is supported by Iiovenen and Sääkslahti’s [81] research in 2014 which reviewed the determinants of FMS in preschool-age children. This previous research also supports the findings of Webster et al. [52], who reported better object control skill performance by boys compared to girls, however, no other studies in this review observed better object control by boys. Object control skills will generally be performed at a lower level of exertion at younger ages, due to longer elements of standing; for example, when catching a ball, a younger child will stand still until they are proficient enough to perform the skill in a moving environment [82]. Therefore, attributing object control skills to increasing MVPA levels is a difficulty faced between the quantity and quality of PA that children are achieving.

In the UK and across several other governments around the globe [3,4,5,83], the recommended amount of MVPA per day for children aged five years and under is at least 60 min, and total PA to be 180 min. Three articles demonstrated children overachieving these guideline levels of MVPA [50,52,53], and Palmer et al. [51], also reported children reaching 22 min of MVPA during 45 min of free play, representing a promising value of 50% MVPA. Despite this, there are conflicting results of sufficient PA being achieved, as reported by Cliff et al. [45], Roscoe et al. [49] and Wasenius et al. [46], where children fell below the national guidelines for the individual studies. Barnett et al. [44] also reported a shortfall in MVPA achievement, however, the children were approaching the recommended guidelines, and these findings are encouraging as they indicate that children have the ability to be sufficiently active, especially if promotion of PA is furthered, helping to highlight that “some is good, more is better” [84]. Duff et al. [47] found children achieving 7.7 min of MVPA/hour during a 3 h school day, representing just 23.1 min of MVPA being achieved in this environment. Similarly, Jones et al. [48] reported just 7% of time at preschool spent in MVPA, resulting in 21 min for a 5 h day of preschool. Educational settings and childcare facilities are seen as facilitators for PA [85,86] and where young children are most active. Therefore, it is unlikely the children in both the Duff et al. [47] and Jones et al. [48] studies would be achieving the full 60 min of MVPA per day when combined with their activity outside these settings. The limitations of the research of both Jones et al. [48] and Duff et al. [47] included the missed opportunities of PA data collection, such as active transport, due to the measurement of PA only during school or preschool hours. Active transport has been reported as an important factor contributing to both PA levels and FMS achievement [87]. Collectively, the current review finds young children to be underachieving recommended levels of PA, especially MVPA, whether this be in an educational environment or inclusive of the home environment. Worryingly, children of older ages continue to struggle to attain PA at guideline levels, especially in developed countries such as the UK [88] and America [89]. Therefore, the promotion and encouragement of PA must be started at as young an age as possible, while continuing to understand the determinants that inhibit and facilitate engagement. Approaches must be centred around children but include support of parents and care givers to achieve the best outcomes to increase the proportion of children performing enough PA [90].

Balance is recognised as a key element of FMS in combination with locomotor and object control skills. The Chief Medical Officer’s guidelines for the UK highlight the need for good balance skills to perform activities such as skating, dancing and gymnastics, which children are encouraged to participate in [91]. Good balance allows children to not only perform better PA, it allows children to perform day to day activities important for physical health, socialisation and education [15,16,17]. The synthesis of balance data in the current review was difficult as there was such a wide and varying array of methodologies, measures and outcomes, and a lack of a standardised approach should be considered as a limitation for this field of research. Fujinaga [65] reported children holding a single-leg stance for an average of over 87 s, while Amelia et al. [63] reported an average of children holding the stance for only 6.67 s. These large differences in time held by the children show why there is a lack of consensus on the expected balance achievement of children this age. Results based around achieving points tended to require children to hold the stance for only up to 10 s, with maximum points achieved for this time [32,62,68]. This approach clearly represents how easily discrepancies between outcomes can occur. With an average of 87 s, it is likely all 105 children in Fujinaga’s [65] research would have achieved mastery in a points-based approach held for 10 s. Future research must focus on the development of a universal balance test or subtest that can be implemented into existing locomotor and object control FMS testing for young children. Considerations such as equipment and materials for measurement need to be considered to make it usable in as many settings as possible by both researchers and child practitioners.

Gender was also found to be an influential factor on balance ability, with girls out-performing boys in all the articles reporting gender differences [34,35,69,71], with the exception of one article [65]. In previous literature, this is commonly attributed to societal gender norms, with girl’s preference and encouragement to take part in activities such as dance and ballet, where single-limb balance may be required frequently [92,93], and thus increase their static balance ability. This highlights the need to ensure that both boys and girls are encouraged to take part in a wide range of activities to increase their exposure to different movement patterns, environments and opportunities. This being said, four articles reported a clear increase in balance ability between children aged 4 and 5 years [34,35,69,74], suggesting that balance ability is affected by maturation to a greater degree than other elements of FMS and MC at these ages.

### 4.2. Measurement

During searching for appropriate literature in the current review, it was apparent other FMS assessments had been used in the research for this age group, aside from the TGMD-2 and -3. Examples included the Movement Assessment Battery for Children, the Bruininks–Oseretsky Test of Motor Proficiency and the Motoriktest für Vier- bis Sechsjährige Kinder. The focus of these movement assessment batteries on identifying motor performance impairments in children [94] or assessing both fine and gross motor development [95] were reasons the authors felt they should not be included in the review. Additionally, a large number of these articles did not measure PA in conjunction with FMS or MC measurement and were discounted even before FMS battery was accounted for. The overwhelming amount of literature used the TGMD-2 or -3, a finding that matches recent reports by both Logan et al. [13] and Klingberg et al. [22]. Despite this, there was still discrepancies in how the assessment batteries were administered between the individual studies, possibly effecting validity and reliability [96]. The difference in both choice and administration of assessment tool is one of the areas leading to a lack of consensus within research to gain a full understanding of the FMS and MC levels achieved in young children, as there is not a universal tool available [23]. Although other FMS assessments are used and found in the literature, the TGMD-2 and -3 are popular due to their focus on locomotor and object control skills specific to sporting performance. The use of these skills in Australia, the USA and the UK are especially common, as society and physical education (PE) within schools is likely to focus on sports performance [97,98,99]. If children have mastery of these skills at an earlier age, then this would be advantageous to the individuals in these environments, because it will likely result in higher PA participation by these children and an opportunity to partake in sport with their peers [77].

ActiGraph accelerometers were used in eighty percent of the articles examining PA, demonstrating a preference in accelerometer brand within childhood research. ActiGraph accelerometers have been consistently reported to be accurate and reliable for younger populations [55,100,101], which makes them a strong choice for objective PA measurement. The popularity of this tool also highlighted the use of the processing programme ActiLife for analysis of accelerometer outputs [44,47,50,51,52], a programme which has been criticised in the literature for its frequency filtration method and the effect on the data collected [102]. Barnett et al. [44] further analysed their results using manual methods within Excel, while Nilsen et al. [53] used Kinesoft software. While the use of accelerometry for PA measurement is clearly popular for collection of PA data with children, and considered the “gold standard”, more recent research has now begun to suggest the use of the raw acceleration output from accelerometers to classify activity and intensity [103,104,105,106,107], in addition to the use of machine learning [108]. These approaches are considered to be more reliable and valid methods, yet researchers may face a number of practical challenges, including having to learn a complex analysis process [109]. The benefits of this method address some of the limitations experienced within this review, especially when cut points, recording Hz and measurement epochs are considered. However, this does not overcome issues in agreement on appropriate wear time, number of days of measurement and what can and cannot be constituted as an accepted day of wear, which was a prevalent issue, even in the ten articles in the current review.

Accelerometer placement at the hip was reported in 90% of the articles, once again showing a clear preference in this element of measurement practice for children. Interestingly, research has shown while there are certainly differences in measurement outcomes at different body placements, including the wrist and hip, these are not significant [100,110]. This suggests wrist placement may be a viable option for wear in the young child population, resulting in higher levels of wear time and compliance [111], possibly resulting in a larger quantity of data. While accelerometry analysis and techniques require improvements to measure light PA (LPA), both sedentary behaviour (SB) and MVPA can be measured accurately [100,107] and, most importantly, these measures are considered the two most important measures when observing young children’s PA. Accelerometry has been criticised for the inability to identify the type of activity being performed [112]. Recent work by Duncan and colleagues [82] has begun to try and establish cut points for certain types of activity and FMS when wearing accelerometers, and this research is an important step as it will allow researchers to identify if children are performing sufficient quality PA and practicing essential FMS, as different types of FMS will be identifiable from data outputs.

In the current review, it is important to note the lack of literature addressing all three areas (FMS, PA and balance) in a single article. Balance is recognised as one of the key components of FMS and MC yet is discounted from a large majority of assessments, hence the need to assess locomotor and object control skills separately from balance in the current review. Both the Movement Assessment Battery for Children and Bruininks–Oseretsky Test of Motor Proficiency batteries assess children’s balance ability, and Eddy et al. [23] reported that the popularity of the Movement Assessment Battery for Children was equivalent to the TGMD, while the Bruininks–Oseretsky Test of Motor Proficiency is to be found with less regularity in the literature. Consistently good reliability and validity was found for the TGMD and the Bruininks–Oseretsky Test of Motor Proficiency, while the Movement Assessment Battery for Children reported weaker levels of these key variables, perhaps providing rationale for why a specific measurement battery may be chosen. However, the number and type of specific skills performed by children during an assessment can be stipulated by individual authors in line with their study aims. This was demonstrated by Nilsen et al. [53], where a balance subset from the “Preschooler Gross Motor Quality Scale” was included, and specific skills including the skip, gallop, slide, dribble, underhand throw and the one-hand and two-hand strike were excluded from the TGMD-3 due to their lack of relevance in the specific society. Despite this, battery protocol guidelines tend to recommend the use of a full protocol for the best outcomes, reliability and validity. A clear difference in the TGMD-2 and -3 to other protocols is the sole assessment of gross motor skills, as the name suggests. As these skills are generally considered to be more important for performing and partaking in regular PA, this could be a key reason for researchers choosing this battery when examining the PA levels of children aged 4–5 years. Future work must ensure the development of a balance skill subtest. Currently, research regularly reports the relationship between locomotor and object control skills with PA, however, the association of balance with MVPA is lacking [27]. Higher balance competency may increase young children’s confidence and ability to perform PA due to increased body control, helping to inform the design of novel interventions.

### 4.3. Strengths and Limitations of the Current Study

Limitations of the study include the specific criteria of only TGMD protocols, as this resulted in possible evidence from other assessment batteries being missed, and this has already been discussed in the “measurement” section. However, the study did allow for any objective measures of PA to be employed and, despite this criterion, all relevant articles were found to use accelerometry over methods such as pedometers. Further to this, richer detail regarding PA during early childhood could have been achieved by reporting all PA levels such as SB, LPA and total PA. This information would inform further research about current behaviours in this age group, such as children choosing to spend more of their time in LPA, and how intervention can be used to increase this to moderate and vigorous levels. Nonetheless, MVPA is recognised as the most important PA level for movement behaviour for young children, which is highlighted in various movement guidelines [3,4,5]. Examining only static balance reduced the evidence available for balance ability in young children and it would be good to consider using both static and dynamic balance measurements within further reviews, as dynamic balance contributes heavily to movement ability [113] and this may also provide a clearer picture on balance ability in this age group. The current study fails to consider the impact of socioeconomic status on the levels of achievement in all three review areas. It is well reported in the literature that socioeconomic status is particularly impactful on the performance of FMS and PA, owing to the relationship of these variables to Newell’s theory of constraints on performance [114]. Factors such as family life, living environment and number of PA opportunities all have been shown to affect performance outcomes [87]. However, the current review collated evidence from global research in one study and, in doing so, it has demonstrated the variation in child populations, and possible causes for differences between cultures. This could help examine how societal changes could create impact in other countries, such as initiatives to improve PA levels.

## 5. Conclusions

To our knowledge, this is the first study to review FMS, objectively measured PA and balance during early childhood (4–5 years old), and thus adds key summary knowledge to the research area. Overall, there is not conclusive evidence for FMS, PA and balance achievement for young children 4–5 years of age. While there is some promising evidence that children have the ability to perform sufficient PA, the FMS competency among the population is worrying and consistently lower than expected. Balance ability remains particularly unclear, with a wide variation in both outcomes and measurement procedures at this age. Future research should therefore focus on establishing a usable and universal balance testing procedure for this age group. Other future work to establish consistently better FMS performance and PA outcomes for children of this age, while reducing the discrepancies between genders through appropriate interventions, is also warranted.

## Appendix A

**Table A1:** PRISMA Checklist.

Section/Topic	#	Checklist Item	Reported on Page
**TITLE**			
Title	1	Identify the report as a systematic review, meta-analysis, or both.	1
**ABSTRACT**			
Structured summary	2	Provide a structured summary including, as applicable: background; objectives; data sources; study eligibility criteria, participants, and interventions; study appraisal and synthesis methods; results; limitations; conclusions and implications of key findings; systematic review registration number.	1
**INTRODUCTION**			
Rationale	3	Describe the rationale for the review in the context of what is already known.	1–3
Objectives	4	Provide an explicit statement of questions being addressed with reference to participants, interventions, comparisons, outcomes, and study design (PICOS).	3
**METHODS**			
Protocol and registration	5	Indicate if a review protocol exists, if and where it can be accessed (e.g., Web address), and, if available, provide registration information including registration number.	3
Eligibility criteria	6	Specify study characteristics (e.g., PICOS, length of follow-up) and report characteristics (e.g., years considered, language, publication status) used as criteria for eligibility, giving rationale.	3–4
Information sources	7	Describe all information sources (e.g., databases with dates of coverage, contact with study authors to identify additional studies) in the search and date last searched.	4
Search	8	Present full electronic search strategy for at least one database, including any limits used, such that it could be repeated.	4
Study selection	9	State the process for selecting studies (i.e., screening, eligibility, included in systematic review, and, if applicable, included in the meta-analysis).	4, 6–8 figures
Data collection process	10	Describe method of data extraction from reports (e.g., piloted forms, independently, in duplicate) and any processes for obtaining and confirming data from investigators.	4
Data items	11	List and define all variables for which data were sought (e.g., PICOS, funding sources) and any assumptions and simplifications made.	4–5
Risk of bias in individual studies	12	Describe methods used for assessing risk of bias of individual studies (including specification of whether this was done at the study or outcome level), and how this information is to be used in any data synthesis.	4
Summary measures	13	State the principal summary measures (e.g., risk ratio, difference in means).	N/A
Synthesis of results	14	Describe the methods of handling data and combining results of studies, if done, including measures of consistency (e.g., I^2^) for each meta-analysis.	5
Risk of bias across studies	15	Specify any assessment of risk of bias that may affect the cumulative evidence (e.g., publication bias, selective reporting within studies).	N/A
Additional analyses	16	Describe methods of additional analyses (e.g., sensitivity or subgroup analyses, meta-regression), if done, indicating which were pre-specified.	N/A
**RESULTS**			
Study selection	17	Give numbers of studies screened, assessed for eligibility, and included in the review, with reasons for exclusions at each stage, ideally with a flow diagram.	9
Study characteristics	18	For each study, present characteristics for which data were extracted (e.g., study size, PICOS, follow-up period) and provide the citations.	9
Risk of bias within studies	19	Present data on risk of bias of each study and, if available, any outcome level assessment (see item 12).	9
Results of individual studies	20	For all outcomes considered (benefits or harms), present, for each study: (a) simple summary data for each intervention group (b) effect estimates and confidence intervals, ideally with a forest plot.	12–19 tables
Synthesis of results	21	Present results of each meta-analysis done, including confidence intervals and measures of consistency.	9–11
Risk of bias across studies	22	Present results of any assessment of risk of bias across studies (see Item 15).	N/A
Additional analysis	23	Give results of additional analyses, if done (e.g., sensitivity or subgroup analyses, meta-regression [see Item 16]).	N/A
**DISCUSSION**			
Summary of evidence	24	Summarize the main findings including the strength of evidence for each main outcome; consider their relevance to key groups (e.g., healthcare providers, users, and policy makers).	20–23
Limitations	25	Discuss limitations at study and outcome level (e.g., risk of bias), and at review-level (e.g., incomplete retrieval of identified research, reporting bias).	23–24
Conclusions	26	Provide a general interpretation of the results in the context of other evidence, and implications for future research.	24
**FUNDING**			
Funding	27	Describe sources of funding for the systematic review and other support (e.g., supply of data); role of funders for the systematic review.	N/A

## Appendix B

**Table A2:** MMAT Quality assessment outcomes.

Author and Year	Score
Barnett et al., 2016 [44]	7
Cliff et al., 2009 [45]	7
Duff et al., 2019 [47]	7
Foweather et al., 2015 [50]	7
Nilsen et al., 2020 [53]	7
Roscoe et al., 2019 [49]	7
Webster et al., 2019 [52]	7
Eshagi et al., 2015 [72]	7
Fujinaga 2008 [65]	7
Moran et al., 2005 [68]	7
Roman et al., 2017 [69]	7
Jones et al., 2011 [48]	7
De Oliveira et al., 2019 [64]	7
An et al., 2009 [73]	6
Jiang et al., 2018 [34]	6
Jung et al., 2017 [66]	6
Stankovic and Radenkovic 2012 [71]	6
Tan et al., 2019 [76]	6
Palmer et al., 2018 [51]	6
Marin 2012 [67]	5
Wasneius et al., 2017 [46]	4
Adamovic et al., 2016 [62]	5
Amelia et al., 2019 [63]	5
Cambier et al., 2001 [74]	5
Condon and Cremin 2014 [75]	7
Guffey et al., 2016 [32]	7
Vanetsanou and Kambas 2011 [35]	7
Zumbrunn et al., 2012 [70]	6
